# What determines health-related quality of life among people living with HIV: an updated review of the literature

**DOI:** 10.1186/2049-3258-72-40

**Published:** 2014-11-17

**Authors:** Sophie Degroote, Dirk Vogelaers, Dominique M Vandijck

**Affiliations:** Department of General Internal Medicine, Infectious Diseases and Psychosomatics, Ghent University Hospital, De Pintelaan 185, 9000 Ghent, Belgium; Department of Public Health, Faculty of Medicine and Health Sciences, Ghent University, De Pintelaan 185, 9000 Ghent, Belgium; Department of Internal Medicine, Faculty of Medicine and Health Sciences, Ghent University, De Pintelaan 185, 9000 Ghent, Belgium; Department of Economics, Faculty of Business Economics, Hasselt University, Agoralaan Building D, 3590 Diepenbeek, Belgium

**Keywords:** HIV, Acquired immunodeficiency syndrome, Quality of life, Epidemiologic factors, Review

## Abstract

**Background:**

As infection with the Human Immunodeficiency Virus (HIV) has evolved to a chronic disease, perceived health-related quality of life (HRQoL) is becoming a prominent and important patient-reported outcome measure in HIV care. Literature discusses different factors influencing HRQoL in this population, however, currently no consensus exists about the main determinants. In this review a clear, up-to-date overview of the determinants influencing HRQOL among people living with HIV is provided.

**Methods:**

All studies published before July 2013 that identified determinants of HRQoL among people living with HIV in high-income countries, were considered in this narrative review. PubMed, Web of Science and The Cochrane Library were consulted using the keywords ‘determinants’, ‘quality of life’, ‘HIV’ and ‘AIDS’. To be included, studies should have reported overall health and/or physical/mental health scores on a validated instrument and performed multivariable regression analyses to identify determinants that independently influence perceived HRQoL.

**Results:**

In total, 49 studies were included for further analysis and they used a variety of HRQoL instruments: Medical Outcomes Study Short Form-36 or variants, Medical Outcomes Study-HIV, HIV Cost and Services Utilization Study measure, Multidimensional Quality of Life Questionnaire, HIV targeted quality of life instrument, Functional Assessment of Human Immunodeficiency Virus Infection, HIV Overview of Problems Evaluation System, EuroQol, Fanning Quality of Life scale, Health Index and PROQOL-HIV. In this review, the discussed determinants were thematically divided into socio-demographic, clinical, psychological and behavioural factors. Employment, immunological status, presence of symptoms, depression, social support and adherence to antiretroviral therapy were most frequently and consistently reported to be associated with HRQoL among people living with HIV.

**Conclusions:**

HRQoL among people living with HIV is influenced by several determinants. These determinants independently, but simultaneously impact perceived HRQoL. Most HRQoL instruments do not capture all key determinants. We recommend that the choice for an instrument should depend on the purpose of the HRQoL assessment.

**Electronic supplementary material:**

The online version of this article (doi:10.1186/2049-3258-72-40) contains supplementary material, which is available to authorized users.

## Background

Since the HAART (highly active antiretroviral therapy) era, HIV has been added to the increasing list of chronic diseases [1, 2]. Still, daily and strict intake of antiretroviral medication is required to achieve or to maintain virological and immunological control [3, 4]. However, these are not the only outcomes that should be taken into account. In chronic diseases, assessing health-related quality of life (HRQoL) has become an integral part of follow-up. HRQoL measurements provide valuable feedback about therapeutic interventions and they are indispensable in cost-effectiveness analyses [5]. HRQoL measures can also identify determinants of HRQoL among people living with HIV (PLHIV), which is vital to maximize HRQoL. Although different studies identified factors associated with HRQoL, there is no consensus about the main determinants. In addition, a variety of HRQoL instruments are currently being used in PLHIV. In this review, we examined determinants reported to be associated with HRQoL in PLHIV and we critically discuss the use of HRQoL instruments.

## Methods

A narrative review was performed. The global search strategy was to search the databases PubMed, Web of Science and The Cochrane Library using the (Mesh)terms ‘determinant(s)’ , ‘quality of life’, ‘HIV’, ’AIDS’ as title words. Articles in this review are 1) investigating HRQoL in HIV-infected (adult) individuals 2) published prior to July 2013, 3) full text available in English, 4) conducted in high-income countries, 5) reporting HRQoL as one total score and/or as a physical and mental score, 6) using a validated HRQoL instrument and 7) applying quantitative methodology and multivariable regression analysis to identify determinants of HRQoL. Titles and abstracts were reviewed to verify those criteria. If all inclusion criteria were fulfilled or if this was still unclear, the manuscript was fully read. In case the full text revealed that not all requirements were present, the manuscript was still excluded from further consideration. Additional literature was obtained through searching references in the manuscripts (snowball method).

A priori, a framework with four categories of determinants was made: socio-demographic, clinical, psychological and behavioural determinants. Those categories were chosen by analogy with research on HRQoL performed in our hospital department. In particular, our research assesses three of the categories through a self-report questionnaire, consisting of a sociodemographic part, a psychological part (assessment of depressive symptoms, social support, satisfaction with care) and a behavioural part (assessment of adherence and substance use). Clinical parameters on the other hand are assessed via the electronic patient record [6]. While reading and interpreting the manuscripts, the categories were further subdivided. Results and conclusions from manuscripts were then allocated to one or more determinants. The review was further elaborated by addressing every determinant separately and rereading all manuscripts that were relevant for that determinant.

In addition to the manuscript, an extensive table is provided in which methodological features (study design, sample, instrument) and main results of all included studies are provided (Additional File 1: Table S1).

## Results

In total, 49 studies were included for further analysis. The instruments used to measure HRQoL were various: Medical Outcomes Study Short Form-36 or variants, Medical Outcomes Study-HIV, HIV Cost and Services Utilization Study measure, Multidimensional Quality of Life Questionnaire, HIV targeted quality of life instrument, Functional Assessment of Human Immunodeficiency Virus Infection, HIV Overview of Problems Evaluation System, EuroQol, Fanning Quality of Life scale, Health Index and PROQOL-HIV (Additional File 1: Table S1).

Determinants of HRQoL were divided into four categories: socio-demographic, clinical, psychological and behavioural factors. For purposes of intelligibility and conciseness, only determinants of overall health outcomes, as well as physical and mental health scores were commented. Factors associated with subscales of HRQoL were not discussed.

### Socio-demographic characteristics

#### Gender

There was no general agreement about gender differences in HRQoL [7]. If differences were found, women mostly reported lower HRQoL than men [8–10]. Different explanations were given: women would be more likely to report their unfavourable physical states than men because men are expected to adopt a more stoic attitude [10, 11], items on physical subscales would be different for men and women [10], and/or women would have more feelings of guilt [12]. Gender differences in HRQoL could also be a consequence of gender differences in mental illnesses. Mood disorders, anxiety disorders and psychosomatic disorders are more prevalent in females, possibly contributing to the HRQoL difference [11]. Controlling for clinical and socio-demographic factors could eliminate gender differences [7].

In contrast, some studies found that females had a better mental health [13] or reported better overall health [14]. Better coping strategies in females and a higher proportion of anxious men in that sample, respectively, were proposed as explanations for those findings. At last, Mrus and colleagues observed no difference towards overall health change between the genders and they suggested that health change, instead of health status, may be a better measure to evaluate HRQoL longitudinally [11].

#### Age

Older age was generally associated with lower physical health [13, 15–19] and with a greater decline in physical health over time [9] most likely due to physical senescence. The relationship with mental health was less distinct. There could be a positive relationship between younger age and better mental health [19] while older age was associated with a decrease in mental health after six months in another study [20]. As most studies were not able to find any association, it seems that mental health is less age-dependent.

#### Family situation

A stable relationship conceivably contributes to a good HRQoL. This was shown, though only for physical health, in two studies [18, 21]. Conflicting results were found with respect to changes in HRQoL over time. In women, being single was associated with an improvement in HRQoL after four months, whereas being married was associated with a decline [22]. Being chronically ill possibly causes more health distress and anxiety in married women, since the illness can interfere with their role as a spouse. However, a stable partner was also found to be positively associated with mental health after one year [9].

Concerning parenthood, a similar picture emerged. Children bring happiness, however, ill parents will be more worried about their health because of them. PLHIV may be more confronted with their constraints through the process of bringing up children [9]. Accordingly, in two studies, parenthood was negatively associated with physical health over time [8, 9].

#### Socio-economic status: education, employment, income

There was a joint consent that socio-economic status has an impact on HRQoL [9, 14, 15, 17, 18, 20, 23–27]. Employment influenced both physical and mental health status, or overall health [14, 17, 23, 24, 28] although the effect on physical health was twice as big [25]. Employment was a positive predictor for good physical health after three to four years [26]. Employment constitutes a big part of the daily life of people. It can provide structure, a social support network, role identity and meaning [29]. The exact association between employment and HRQoL, however, remained unproven. Probably this is a bi-directional relationship: good HRQoL could be a requirement to be able to work (selection hypothesis), or work could be a source of well-being (causation hypothesis) [25, 27].

There were indications for an association between financial situation and HRQoL [15, 18, 23]. A low versus middle- or high income was associated with a lower mental and overall health [23]. Yet, income may be not the best measure to assess financial situation. Data concerning expenditures, family composition, financial insecurity and financial worries could provide useful additional information [30].

Education has also been linked to HRQoL and the relationship would be linear (i.e. higher education associated with higher HRQoL) [23]. An education of less than five years was negatively associated with mental health after six months [20] and with physical health change after 12 months [9]. It is possible that a lower education is a proxy of a lower socio-economic status in general, but it could also represent a poorer ability to understand the therapy recommendations [20]. This leads to less informed, less involved and less empowered patients which affects their HRQoL [31, 32].

### Clinical and disease-related factors

#### Virological and immunological status

A better virological status was associated with a better overall [33], physical [14, 18, 34] and mental health [14], although a reasonable amount of studies did not find an (independent) association [8, 13, 15, 20, 27].

On the other hand, the relationship between immunological status and HRQoL was better corroborated by scientific evidence: a higher CD4-cell count was associated with a better physical health [15, 16, 20, 21, 34, 35]. It was also a predictor for better physical health scores on follow-up measurements [20, 36, 37]. However, physical health improved more in patients who started ART at CD4 <200 cells/μl than in patients with 200–350 cells/μl or >350 cells/μl, because their physical health at baseline was worse [38]. Current guidelines recommend nonetheless to start ART early (i.e. 350–500 celles/μl), because there is evidence that this results in reduced progression to AIDS and reduced mortality [39]. In another study, CD4-count was negatively associated with HRQoL at baseline, but positively after 12 months [40]. It is possible that PLHIV with a high CD4-count at baseline wanted to go back to their excellent health and therefore considered their actual health status as dissatisfying. Over time, PLHIV develop other perceptions and expectations about HRQoL and may consider their health status acceptable [40].

Three studies have found an additional relationship with mental health. A lower CD4 cell count was then associated with a lower mental health score (MHS) (cross-sectional and at 12 months) [9, 18, 35]. This could be due to the faster disease progression in PLHIV with low CD4-counts, leading to distress [9].

#### Staging and time since diagnosis

Regarding disease stage, a clear effect on physical health has been established: as HIV evolves to a further stage, physical health diminishes. The poorest physical health is seen in people with AIDS [18, 23, 25] and AIDS negatively influenced future physical health in follow-up studies [9, 26, 36]. In contrast, the effects on mental health were variable. A longer time since diagnosis was associated with both lower [14] and higher mental health [25]. A lower mental health over time could arise in cases where longitudinal follow-up resulted in a successful control of HIV. PLHIV may then experience difficulties to report mental problems because they are expected to feel fortunate because of their good health [14]. On the other hand, a longer duration could facilitate the development of effective coping strategies which could enhance mental health [40]. For health care providers, it is important to monitor mental health continuously, irrespective of the physical condition of the patient.

#### ART

The effect of ART on HRQoL has been described as a balance between reduced HIV-related symptoms and better life-expectancy on the one hand and medication side-effects on the other hand [41]. In people with an acceptable health status prior to ART-initiation, these side-effects could even overrule the benefits [21]. Encountering few ART-related side-effects contributed to normal physical and mental health [21]. Sexual dysfunction is an example of a side-effect – although frequently seen without iatrogenic cause – which was found to have a significant impact on HRQoL [42].

Recent studies mainly showed a positive effect of ART on HRQoL. This could be due to the continuous improvements in ART leading to less side-effects and to possibilities of combination preparations [43, 44]. ART treatment was independently associated with a higher physical health [17] and interruption of ART treatment was associated with lower mental health [15]. In some studies, no HRQoL-differences between ART-treated and non-treated PLHIV were observed [8, 14, 18], or only at bivariate level [20]. Maybe non-treated PLHIV had a better health, through which treatment was (still) not required [14].

#### Presence of symptoms

The negative influence of HIV-related symptoms on HRQoL was supported by scientific evidence and generally, a relationship with both physical and mental health was found [20, 23, 25, 26, 28, 45, 46]. Long-term studies found the same results [9, 36]. In the HIV Cost and Services Utilisation Study, there was an inverse linear relation between number of symptoms and SF-36 scores. Each additional symptom (out of 13), was associated with a nearly 1.5-point decrease in physical and mental health [23]. Furthermore, symptom status was a stronger predictor for HRQoL than functional status, health perceptions, age, sex, biological and physiological markers [47].

Lipodystrophy was found to be an influential HIV-related symptom [48]. It affected both physical and mental health [48] and was associated with lower mental health after one year [9]. In time trade-off preference measurements, PLHIV would even give up a life year to avoid lipodystrophy [49]. It was although possible that HRQoL was affected only in PLHIV perceiving a moderate to severe body alteration [50]. Lipodystrophy is a highly visible symptom, and it could also affect the self-esteem and social contacts of PLHIV [48]. Similar negative effects of weight loss have been reported [14].

#### Comorbidity

A negative effect of comorbidity burden on HRQoL, especially on physical health, was supported by cross-sectional studies [15, 51] and a longitudinal study [40]. Regarding specific comorbidity, comorbid hepatitis B was associated with a lower physical health and hepatitis C negatively influenced physical health after one year [9]. On the other hand, HRQoL was the same among PLHIV, people infected with hepatitis C and people infected with both HIV and hepatitis C [17]. PLHIV with a GB virus C infection reported even a better quality of life than PLHIV without GB virus C infection [52]. Authors reasoned that patients’ HRQoL could be more affected by socio-economic and psychological factors than physical illnesses as HIV or hepatitis C, as the latter have become manageable diseases [17], or they may be a more favourable course of HIV infection in patients with GB virus C infection [52].

### Psychological factors

#### Depression and anxiety

Depression strongly interferes with daily life and the negative impact on HRQoL was obvious [8, 46, 53, 54]. Some authors did only find an association with mental health, and not with physical health [9, 15, 16, 55] although depression can also cause physical problems (e.g. less appetite, sleep disorders…). Not only the presence, but also a history of depression was associated with poorer HRQoL [17]. Depression also mediated the relationships between coping styles, social support and HRQoL [16, 53], which will be discussed further on. The prevalence of anxiety disorders in PLHIV is higher than in the general population and it is known that anxiety is associated with depressive symptoms [14, 56]. Anxiety was found to be associated with a diminished physical and mental health [18].

#### Coping styles, locus of control and religion

Coping styles are highly relevant in PLHIV, looking for example at the stress resulting from HIV diagnosis and treatment as well as multiple possible other stressors [53, 57]. Studies examining the effect of coping styles on HRQoL have often found that the effect was indirect, namely via the intermediate determinant depression [16, 53]. An active coping style has also been found to be directly associated with a better HRQoL [35, 40]. People adopting an active coping style undertake actions to enhance their current state and this coping style is generally thought to be effective in reducing stress [58] and the impact of life stressors on mental health [19]. Coping styles are also associated with health locus of control (HLOC). People with an internal HLOC believe that health outcomes result directly from one’s own behaviour whereas people with an external HLOC believe that others– other persons, fate, or luck– determine the outcome [59]. An internal HLOC was a predictor of a better physical health and a powerful others HLOC predicted a lower mental health [26]. However, somewhat contrary to that, a higher level of spirituality/religion (which could be associated with an external HLOC) has been found to be correlated with the perception of a better life then before the HIV diagnosis. It seems that religious/spiritual coping could also be an effective coping style [60]. Nevertheless, religious coping could be ineffective too, by arousing feelings of punishment by God, or being ostracised [61].

#### Social support

Social support could directly influence health outcomes, or it could serve as a buffer to reduce the influence of stressors on health outcomes [62]. Many studies have found positive relations between the presence of social support and both physical and mental health [13, 25, 53]. Furthermore, a higher social support at baseline was predictive for a better mental health after 12 months [40]. In the study of Jia et al. [53], social support had a mainly indirect role in HRQoL, mediated by depression. This suggests that enhancing social support could reduce depressive symptoms, which then could improve HRQoL.

It seemed that the more people PLHIV have to talk to (i.e. providing social support), the better the mental health was [15]. By studying different types of social support (emotional and tangible social support), Friedland et al. were able to distinguish different patterns [63]. Emotional social support (e.g. empathy, affection, caring) positively influenced HRQoL and tangible social support (e.g. financial and material assistance) negatively influenced HRQoL. People who received more tangible social support perceived themselves as more ill and as having a lower HRQoL [63]. Associations between social support and HRQoL over time seemed to vary. Possibly, social support becomes more important at difficult times [64].

#### Neuropsychological status

Cognitive problems lead to difficulties in every day practices, such as remembering names of new people, concentrating during a conversation or finding solutions for unexpected problems [65]. These will most probably affect HRQoL. In a study in HIV positive women, neurocognitive performance together with emotional distress accounted for most of the variance of HRQoL [66]. The validation study of the Quality of Well-being scale for HIV patients also revealed a significant influence of neurocognitive impairment [54]. In another study, neurocognitive impairment reduced physical health, but not mental health [55].

#### Other (health care, disclosure, stigma)

The way PLHIV perceive health care service has shown to be important for the patient’s HRQoL [18, 20]. Poor satisfaction with information from health care providers was independently associated with a lower mental health [20]. Inadequate communication with the physician could lead to misunderstanding, uncertainty and a lack of trust in the caregiver [20]. Therefore, communication is considered one of the core competencies of physicians [67]. Some PLHIV even experience rejection by the medical staff, especially people infected through drug use. Wrong attitudes could hinder a good physician-patient relationship, which is crucial for physical HRQoL [18]. Experiencing stigma negatively influenced HRQoL [28] and discrimination from family, friends or at work negatively influenced mental health [18]. Disclosing seropositive status was found to have negative effects on both physical and mental health [18]. The latter relationship, however, remained unclear, because disclosure could also be beneficial for the patient’s mental and physical health (mainly through social support) [68]. However it could potentially be related to the fact that people with worse HRQoL have to disclose their seropositive status because of the symptoms, and may feel forced to do so instead of voluntarily doing so. PLHIV should be prepared and supported by health care professionals before their decision whether or not to disclose and before they are confronted with stigma and discrimination, in order to be able to better cope with these difficulties [46].

### Behavioural factors

#### Smoking

The relationships between smoking and HRQoL was not extensively studied yet [69]. Two studies suggested that smoking negatively influenced HRQoL [16, 69]. Among HIV-positive veterans, current smokers had the lowest HRQoL as compared to former and never smokers [69]. Current smokers were more at risk for pulmonary diseases and respiratory symptoms, which may affect their physical health [69]. However, smoking was also found to be negatively associated with mental health [16]. Psychiatric comorbidity was found to be highly prevalent in smokers – or the other way around, since patients reported that smoking helps them cope with depressive symptoms and anxiety [70].

#### Alcohol use

The effect of alcohol use on HRQoL in PLHIV was scarcely or only superficially studied [71]. In none of those studies among PLHIV, an independent association between alcohol use and HRQoL was found [8, 18, 71] except for one. In that study, not using alcohol was a predictor for a lower physical health [15]. A causal relationship between light to moderate alcohol use and a better physical HRQoL is, however, not very plausible. Light to moderate alcohol use could rather be seen as an expression of risk behaviour for which a certain degree of functional status is required [15].

#### Drug use

Quality of life in HIV-positive drug users has been relatively more studied than in tobacco and alcohol users, because IDU is a risk factor for HIV-transmission. Current drug use was associated with lower mental health [15, 71, 72]. HRQoL in former users of illicit drugs was not worse than in persons who never used drugs [71]. For IDU the same was found: being an IDU was associated with a lower physical health [13], whereas being a former IDU was associated with a better physical and mental health [21]. Impact of drug use on HRQoL could be direct, via the physical deterioration caused by the drugs [13] or indirect, via associated psychiatric disorders [72].

#### Adherence

In a review of twelve studies considering the relationship between adherence to ART and HRQoL, ten showed an association with HRQoL [73]. Three of them applied a prospective design and were thus more indicative for a causal relationship [73]. More adherent PLHIV probably had better virological and immunological outcomes and could therefore have a better HRQoL.

Reporting concerns and difficulties about taking medication, was associated with poor mental health [13, 74]. These difficulties could originate in a low self-efficacy, a high pill burden, difficulties to incorporate the medication moments in daily activities, experiencing side-effects…

#### Life style and sexual risk behaviour

Not surprisingly, physical activity was associated with a better physical health [16, 75, 76]. Healthy nutrition and a good stress management were associated with both physical and mental health [75]. Associations between unsafe sex and HRQoL were contradictory: in one study, there was no relationship to either physical or mental health and in another study [75], having unsafe sex with casual partners was associated with poor mental health [77].

## Discussion

This review presents an overview of socio-demographic, clinical, psychological and behavioural factors associated with HRQoL in PLHIV. Many determinants have been found, and it is not clear which determinants are the strongest predictors for HRQoL. Nonetheless, there seems to be a consensus about the effect of socio-economic status, immunological status, presence of symptoms, (psychiatric) comorbidity, social support and adherence to ART.

These partly correspond to determinants of HRQoL of people living with other diseases. In patients with Crohn’s disease, epilepsy, Parkinson’s disease and aneurysmal subarachnoid haemorrhage, advanced disease stage (cfr. immunological status) was found to be predictive for impaired HRQoL [78–81]. Disability (cfr. presence of symptoms) was a determinant of HRQoL in patients with Crohn’s disease, Parkinson’s disease and aneurysmal subarachnoid haemorrhage [78–80]. Depression was negatively associated with HRQoL in patients with epilepsy, Parkinson’s disease and aneurysmal subarachnoid haemorrhage [79–81]. Presence of comorbidities was associated with lower HRQoL in patients with epilepsy, lung cancer and in long-term survivors of colorectal cancer [81–83].

Literature about HRQoL in PLHIV consisted mostly of cross-sectional studies, however some longitudinal and interventional studies were found as well. Overall, there was a great diversity of instruments to measure HRQoL in PLHIV. Both generic (regularly SF-36) and disease-specific (MOS-HIV, HCSUS, HAT-QOL …) instruments were used (see Table 1). It is impossible to point out the best HRQoL instrument for PLHIV, since that choice mainly depends on the purpose of the assessment. For example, cost-effectiveness analyses require a HRQoL instrument that provides an index score while clinical routine management requires an instrument that provides information about HIV-specific aspects influencing HRQoL in PLHIV. Up till now, however, many HIV-specific instruments omit some of those aspects (e.g. stigma, coping and resilience, body image…) and literature concerning those determinants is rather scarce [35, 84, 85]. Instruments developed in cooperation with PLHIV (e.g. FAHI, HAT-QOL, HOPES, PROQOL) try to minimize that limitation. HRQoL instruments in PLHIV have been compared and discussed by many researchers, mainly by means of two-by-two comparisons in a selected population. Two studies provide a more general overview: Clayson et al. brought three generic (SF-36, EuroQol and Health Utilities Index) and two HIV-specific (MOS-HIV and FAHI) instruments forward as appropriate for use in clinical trials [86]. Their conclusions were based upon assessment of content, practicality and psychometric properties. Following their criteria, generic instruments should also be able to provide preference-based index scores and they should have normative population data. Grossman et al. suggest the SF-12 and MOS-HIV as the most appropriate tools [87]. They made a trade-off between the brevity of the instrument and the usefulness of the information provided.

**Table 1:** **Overview of HRQoL instruments used in the reviewed manuscripts**

Name of the instrument	Generic or HIV-specific instrument	Measurements
SF-36 (Medical Outcomes Study Short Form-36 or variants (SF-12, SF-8)) [88]	Generic	8 subscales
		Physical health summary
		Mental health summary
MOS-HIV (Medical Outcomes Study-HIV) [89]	HIV-specific	11 subscales
		Physical health score
		Mental health score
HCSUS (HIV Cost and Services Utilization Study tool) [90]	HIV-specific	10 subscales
		Physical health
		Mental health
		Overall health
MQoL-HIV (Multidimensional Quality of Life Questionnaire for people with HIV/AIDS) [91]	HIV-specific	10 subscales
		Global score
HAT-QOL (HIV targeted quality of life instrument) [92]	HIV-specific	9 subscales
		Global score
FAHI (Functional Assessment of Human Immunodeficiency Virus Infection) [93]	HIV-specific	4 subscales:
		Physical well-being
		Emotional well-being
		(Social well-being)*
		(Functional well-being)*
HOPES (HIV Overview of Problems Evaluation System) [94]	HIV-specific	6 subscales
		Global score
EQ (EuroQol) [95]	Generic	Global score (utility)
Fanning Quality of Life Scale [96]	HIV-specific	Global score
HI (Health Index) [97, 98]	Generic	Physical well-being
		Mental well-being
PROQOL [99]	HIV-specific	7 subscales
		(+ 1 on treatment impact)
		Global score

*Determinants of the FAHI-subscales ‘social well-being’ and ‘functional well-being’ were not considered in this review.

The heterogeneity of HRQoL instruments was also present in the types and numbers of possible determinants that were analysed. Moreover, the detail by which these determinants were studied, varied from one single question to a whole battery of tests and interviews. Although this makes it difficult to compare results, it permits to 1) state that a certain factor is associated with HRQoL, independently of many other variables and 2) describe this effect more profoundly.

This is a narrative review, and this method has several limitations. The literature search was not integrally documented, and is as such impossible to reproduce and the inclusion of the studies could be subject to personal bias. Nonetheless, we reported transparently about the search method and aimed at composing a review as complete and as consistent as possible. The latter is also the reason for including only papers that used quantitative, multivariable regression analyses. Thereby, we do not want to detract from the great value of papers using other methodologies (especially qualitative research).

## Conclusion

HRQoL in PLHIV is influenced by many concurrent factors. From the evidence studied here we can conclude that there is a consensus about the influence of socio-economic status, immunological status, presence of symptoms, (psychiatric) comorbidity, social support and adherence to ART. Various instruments can be used to assess HRQoL in PLHIV, however, all have limitations. The choice of instrument should depend on the purpose of the HRQoL assessment.

## Electronic supplementary material

Additional File 1: Table S1: Overview of the included articles. (DOCX 35 KB)

Below are the links to the authors’ original submitted files for images.

Authors’ original file for figure 1
