# Supplementary material for: What determines health-related quality of life among people living with HIV: an updated review of the literature
Source: Arch Public Health. 2014 Nov 17;72:40. doi: 10.1186/2049-3258-72-40 (PMC4323115; doi:10.1186/2049-3258-72-40)
Supplement: Supplementary file 1 — Additional File 1: Table S1: Overview of the included articles. (DOCX 35 KB) [file 13690_2014_5055_MOESM1_ESM.docx]

**Additional File 1: Table S1: Overview of the included articles.**

| **Study** | **Country, setting** | **Sample** | **Measures** | **Study design** | **Main findings** |
| --- | --- | --- | --- | --- | --- |
| Airoldi et al  2010 | Italy, multicenter | 202  77.4% m, mean 45.8y, 39.2% homosexual, 15.6 IDU, 556 mean CD4, all undetectable, all on ART | SF-36 | Longitudinal (Baseline, 1,2,4,6 months) | Better HRQoL after switch to single tablet regimen |
| Armon et al  2012 | USA, monocenter | 159  90.6% males, median 43 (39-49)y, 68.6% white, 16.4% black, 13.2% Hispanic, 18.9% IDU, 62.3% homosexual, 92.5% ART, 1.4 (IQR 1-2) VL, 455 (IQR 275-735) CD4 | MOS-HIV  (+ Brief COPE Inventory) | Cross-sectional | MH: public or no insurance +, mental illness -, number of non-HIV medications taken -, CD4 cell count +, maladaptive coping behaviors -, adaptive coping behaviors. / PH: maladaptive coping behaviors - |
| Blalock et al  2002 | USA, monocenter | 200  75% m, median 40 y, 23% Caucasian, 70% African American, 5% Hispanic, 75% HAART, all CD4 < 200 | MOS-30  (+ Brief Symptom Inventory + Structured Clinical Interview for DSM-IV non-patient version HIV) | Cross-sectional | HRQoL: employment + |
| Bouhnik et al  2006 | France, multicenter (102) | 591  all males, all homosexual, 43y mean, 12% ≤ 200 CD4, 63% undetectable VL (<400), 81% on ART | SF-36 | Cross-sectional | MH: unsafe sex - |
| Briongos-Figuero et al  2011 | Spain, monocenter | 150  74.7% m, 44.3 ± 8.3 y, 8% immigrants, 18.7% homosexual, 38% IDU, 9.3% CD4<200, 23.3% undetectable, 84% on ART | MOS-HIV | Cross-sectional | PH: female gender - and hospitalization -, parenthood -, depression - MH: depression -, chronic hepatitis C - |
| Burgoyne et al  2004 | Canada, monocenter | 41  85% m, mean 38.8y, 315 mean CD4, 3.9 mean VL, 83% on ART | SF-36 (+ MOS Social Support Survey) | Longitudinal (Baseline, 2, 4 years) | Stability in quality of life over 4 years  HRQoL: perceived social support +, symptoms -, and immunologic status +, virological status + |
| Call et al  2000 | USA, monocenter | 158  87% m, 39 ± 8.6y, 64% Caucasian, 73% homosexual, 8% IDU, 69542 median VL , 144 median CD4, 75% on ART | SF-36 | Cross-sectional | PH: viral load -, after controlling for CD4 cell count |
| Cederfjall et al  2001 | Sweden, monocenter | 189  70.9% m, 37-42y, 41.3% homosexual, 15.9% IDU, 81% on ART, 18.5% <200 CD4 | Health Index  (+ HIV symptom scale, Sense Of Coherence scale, Well-being Scale, Interview Schedule for Social Interaction) | Cross-sectional | HRQoL: Sense Of Coherence + |
| Corless et al  2013 | USA, Puerto Rica, Africa, multicenter (13 clinics) | 702  61.6% m, 43.4 ± 9.3y, 23.4% white, 33% African American, 30.7% Hispanic, 71.4% on ART | HAT-QoL (+ Stressful Life Events Scale + Revised Sign and Symptom Checklist) | Cross-sectional | HRQoL: medication adherence concerns -, total physical and psychological symptoms - |
| Crothers et al  2005 | USA, multicenter (3) | 867  99% males, mean 49-53y, 33-39% white, 50-55% African American, 319-377 mean CD4, 2.3-3.1 mean VL, 78-84% on ART | SF-12 | Cross-sectional | PH: current smoking - |
| Fleming et al  2004 | USA  (1 center) and Jamaica (1 center) | 299  (53 HIV, 110 HCV, 136 HIV/HCV) 69-77% males, mean ± 45 y, 8% MSM in HIV/HCV and HCV - 53% in HIV, 73-89% IDU in HIV/HCV and HCV - 2% HIV, 17-22% <200 CD4, 46-54% <50 copies VL | SF-36 | Cross-sectional | HRQOL for the group of HIV/HCV coinfected patients was similar to that of HRQOL in patients with either HCV or HIV alone.  PH: age -, unemployment -, no ART use -  MH: being immigrant -, IDU -, functional status +, depression - |
| Friedland et al  1996 | Canada, multicenter (5) | 120  89.2% m, mean 37y, 74.8% homosexuals, 13% IDU | Fanning QoL scale (+ Evans QoL questionnaire + Index of Well-being + Interpersonal Support Evaluation List + Social Support Inventory for People with AIDS + Ways of Coping Questionnaire + Recent Life Changes Questionnaire (health status) + Symptom Checklist) | Cross-sectional | HRQoL: income -, perception-oriented coping +, tangible social support -emotional social support + |
| Gibson et al  2011 | Canada, multicenter | 758  82% m, 47 ±10y, 64% Caucasian, 67% homo/bisexual, 74% undetectable VL, 58% <500 CD4 | SF-36  (+ National Population Health Survey (NPH) Stress Questionnaire + Pearlin Mastery Scale + Medical Outcomes Study Social Support Survey (MOS-SSS) + Brief COPE) | Cross-sectional | PH, MH: number of stressors - |
| Hays et al  2000 | USA, multicenter (unknown) | 2864  77% m, 39 ± 9 y, 49% white, 33% African American, 15% Hispanic, 49% homosexual, 24% IDU | HCSUS tool (based on SF-36) | Cross-sectional | PH: income +, unemployment -, AIDS-, symptoms –  MH: age +, education +, income +, unemployment -, CD4 cell count +, symptoms - |
| Herrmann et al  2013 | Australia, monocenter | 102  85.3% m, 46 (37-53.8)y, 78.4% Caucasian, 52.9% homosexual, 5.9% IDU, 75.5% undetectable VL, 530(376-733) CD4 cell count, 85.3% on ART | PROQOL-HIV | Cross-sectional | HRQoL: age>45y +, unemployment -, depression -, sexual dysfunction -, gastrointestinal symptoms -, malaise -, morphological symptoms -, other symptoms - |
| Holzemer et al  2009 | USA, Puerto Rica, Africa, multicenter (14 clinics) | 726  54.6% m, 42.7 ± 9.5y, 21.0% white, 29.7% African American, 27.6% Hispanic, 15.4% African Continent, 40.9% AIDS, 70% on ART | HIV/AIDS Targeted Quality of Life instrument  (+ Revised Sign and Symptom Check-List for Persons with HIV Disease + Perceived Stigma Scale + CES-D) | Cross-sectional | HRQoL: symptoms -, depression -, stigma - |
| Jia et al  2004 | USA, multicenter (3) | 226  all males, 45.7 ± 8.7y, 54.9% white, 31.9% homosexual, 8% IDU, 25.3% CD4<200 | HCSUS tool (+ Social Support Appraisal Scale + Coping with HIV questionnaire + CES-D + Patient Medication Adherence Questionnaire-7 + Charlson Co-morbidity Index) | Cross-sectional | PH, MH and overall HRQoL: social support +, depressive symptoms - MH and overall HRQoL: effective coping +  The effects of social support and coping were mainly through the intermediate factor of depressive symptoms. |
| Jia et al  2005 | USA, multicenter (3) | 197  all males, 45.8 ±8.4 y, 55.8% white, mean CD4 426 ± 284 | HCSUS tool  (+ Coping with HIV questionnaire + Social Support Appraisals Scale + CES-D) | Longitudinal (Baseline, 12 months) | PH 12m: CD4 cell count +  MH: depression – |
| Jia et al  2007 | USA, multicenter (3) | 197  all males, 45.8 ± 8.4y, 55.8% white, 426 ± 284 | HCSUS tool  (+ Coping with HIV questionnaire + Social Support Appraisals Scale + CES-D) | Longitudinal (Baseline, 12 months) | PH: interaction effect of CD4 and time (at baseline -, at 12m +), comorbidity –  MH: social support + |
| Korthuis et al  2008 | USA, multicenter (14) | 951  68% m, 31% white, 51% black, 14% Hispanic, 69% on ART | HCSUS tool | Cross-sectional | PH and MH: current illicit drug use –  MH appeared to be more severely impacted by illicit drug use than PH. |
| Kowal et al  2008 | Canada, monocenter | 97  84% m, 39.4 ± 8.7y, , 71% Caucasian, 368.9 ± 199.9 CD4 count | SF-36  (+ Center for Epidemiological Studies-Depression Scale (CES-D) + Coping Inventory for Stressful Situations) | Cross-sectional | PH: physical activity +, CD4 cell count +  MH: smoking -, depressive symptoms - / Depressive symptoms mediated the relationship between coping styles and MH. |
| Liu et al  2006 (ref 15) | USA, multicenter (4) | 636  all males, 43.3y (IQR 39.0-48.2), 84% white | SF-36 | Longitudinal (Baseline, every 6 months) | PH: age -, socioeconomic status +, number male sexual partners -, no alcohol drinking -, more advanced HIV disease stage-  MH: outpatient visits -, antiretroviral drug interruption -, recreational drug use -, social support + |
| Liu et al  2006 (ref 41) | USA, multicenter (4) | 68  all males, mean 43y, 84% white | SF-36 | Longitudinal (Pre-seroconversion, post-seroconversion, post-initiation ART) | PH decreased significantly after seroconversion and after starting HAART < 2 years . After 2 years of HAART use, PH was relatively stable, but further decreased after 4 years on HAART  PH: age -, income +  MH declined, though not significantly, after seroconversion and then started to increase after longer HAART use. MH: depression - |
| Lorenz et al  2006 | USA, multicenter (unknown) | 2267  70.7% m, 51% white, 31.2% African American, 14.4% Hispanic, 47.2% homosexual, 23.4% IDU | 2 questions SF-36:  ‘‘How would you rate your current health, overall?’’  ‘‘How would you rate your quality of life, overall?” | Longitudinal (Baseline, 18 months) | HRQoL increases for people with decreasing number of symptoms, HRQoL decreases for increasing or unchanged number of symptoms |
| Mrus et al  2005 | USA, multicenter | 1178  82.9% m, 36.2 (±0.6)- 39.2 (±0.3) y, 30-56% white, 15-48% black, 17-21% Hispanic, all CD4 <200 | AIDS Clinical Trials Group QoL 601-602 Health Survey (adaptation of SF-21) | Longitudinal (baseline, 24 weeks, 40 weeks) | Women reported poorer HRQoL than men in the domains of physical functioning, pain, and energy/fatigue. Changes in domain scores over time and in response to treatment did not differ. |
| Murri et al  1997 | Italy, monocenter | 213  66.6% m, mean 36y, 25.4% homo/bisexual, 43.6% IDU, 38% <200 CD4, 71.8% on ART | MOS-HIV | Cross-sectional | HRQoL: symptoms -, number of daily pills - |
| Murri et al  2003 | Italy, multicenter | 809  68.1% m, 36 (IQR 32-40)y, 14.7% homosexual, 48.2% IDU, 38.1% CD4<200, 39.7% undetectable VL, 89.9% on ART | MOS-HIV | Longitudinal (Baseline, 6 months) | PH baseline: CD4 cell count +, hospitalization -, symptoms –  MH baseline: hospitalization -, symptoms - and satisfaction with information from providers +  PH 6 months: more advanced stage of HIV infection -, baseline CD4 cell count +, PH + and symptom score –  MH 6 months: age -, baseline MH +, symptom score - and education + |
| Nieuwkerk et al  2007 | The Netherlands, multicenter (22) | 256  3 groups: <200 (n = 106), 201-350 (n = 65), and >350 CD4 (n = 68)  96-88-93% m, 39.4 ± 7.7-40.3 ± 10.2-40.4 ± 8.4 y. 70 (IQR 20-140)-271 (IQR 230-320)-450 (IQR 390-635) CD4, 5.2 (IQR 4.8-5.5)-5.0 (4.5-5.3)-4.8 (4.4-5.0) VL | MOS-HIV | Longitudinal (Baseline, every 6 months) | PH improved more among patients who started HAART with <200 CD4 than among those with >200  No difference on physical QoL between patients who started HAART with 201 to 350 CD4 cells/mL versus those with >350 CD4 cells/mL  No difference in impact on mental QoL between the 3 groups. |
| Osowiecki et al  2000 | USA, monocenter | 36  all females, 37.9 ± 6.7y, 428.2 ± 225.2 CD4 | MQoL HIV (+ Profile of Mood States + neurocognitive measures) | Cross-sectional | HRQoL: neurocognitive performance +, severity of emotional distress - |
| Perez et al  2005 | Spain, multicenter (4) | 320  73.4% m, mean age 39.7 (±8.6), 46.9% IDU, 471.5 ±291.1 mean CD4, 61.6% undetectable VL, all on ART | MOS-HIV  (+ Duke-UNC-11 Functional Social Support Questionnaire + GHQ-28 - psychiatric morbidity) | Cross-sectional | PH: psychological morbidity -, social support +, intravenous drug use -, hepatitis B -, CD4 count +, manual job +, older age –  MH: social support +, only one additional pill +, difficulty in taking the medication -, female gender + |
| Perez et al  2009 | Spain, multicenter (4) | 540  73.4%m, 38-41y, 32.6-52.7% IDU, 65.1-68.4% undetectable VL, 448.6-544.5 average CD4, all on ART | MOS-HIV  (+ Duke-UNC-11 Social Support Questionnaire + Goldberg’s questionnaire *(psychiatric morbidity)* | Cross-sectional | PH, MH: no differences among genders after controlling for clinical and demographic variables |
| Preau et al  2005 | France, multicenter (47) | 309, 80% m, 38.8 ± 9.2 y, 14% IDU, 26% ≤ 200 CD4, 4.2 ± 1, 62% on ART | SF-36  (+ Multidimensional Health Locus of Control Scale) | Longitudinal (Baseline, 44 months) | Internal HLOC beliefs were associated with improved physical HRQoL. |
| Preau et al  2007 (ref 18) | France, multicenter (102) | 2235  73.6% m, 42.4 ± 9.4 y, 15.5% immigrants, 45.1% homo/bisexual, 16.1% IDU, 470 (IQR 314–644) CD4, 80% on ART | SF-36  (+ Hospital Anxiety and Depression Scale) | Cross-sectional | PH: < 47 years -, stable relationship +, financial difficulties -, uncomfortable housing conditions -, detectable VL -, AIDS-defining events -, adverse HIV treatment reactions -, rejection by medical staff -, disclosure of seropositivity -, HAD scores -, psychoactive drugs –  MH: financial precariousness -, CD4 count +, adverse treatment reactions -, disclosure of seropositivity, discrimination -, HAD scores -, psychoactive drugs - |
| Preau et al  2007 (ref 21) | France, multicenter (unknown) | 243  67% m, 37-38 (35-41)y, all IDU, 7% CD4 <200, 80% ART | SF-12  (+ Life Experience Survey Scale + CES-D) | Longitudinal (Baseline, 42 months) | PH: age -, stable partner +, being a former IDU +, CD4 cell count > 500 +, financial-related NLE -, HAART self-reported side effects –  MH: social support from partner +, being a former IDU +, violence-related NLE -, HAART-related side effects - |
| Protopropescu  et al  2007 | France, multicenter (unknown) | 1000  77.3% m, 37.1 (±0.3) y, 74.6% born in EU, 43% homosexual, 17.4% IDU, median CD4 293 (IQR 154-440), all on ART | SF-36 | Longitudinal (baseline, 1 year, 5 years) | Positive change in PCS and MCS over the first 12 months, stable trend in the maintenance phase of the treatment, from M12 to M60  PH and MH: CD4 count +, side-effects -, poor housing conditions - PH: older age - higher education +, parenthood -, more advanced disease stage -, HCV coinfection -, homosexual transmission +  MH male gender +, stable partner +, IDU -, symptoms of lipodystrophy -, antiretroviral naivety - |
| Rajagopalan et al  2008 | USA (unknown) | 1124  78.7% m, median 40-49y | SF-8 | Cross-sectional | PH and MH: lipoatrophy - |
| Rao et al  2007 | USA, multicenter (4) | 273  87% m, 39 (±8.7) y, 59% Hispanic, 61% ≤ 200 CD4, 70% on ART | FAHI  (+ Woodcock Language Proficiency Battery– Passage Comprehension subtest) | Cross-sectional | HRQoL (functional health status): female gender - |
| Rodriguez-Penney et al  2013 | USA, multicenter (unknown) | 141  81-82% m | SF-36  (+ Charlson Co-morbidity Index (CCI) + Reading subtest of the Weschler Test of Adult Reading + HIV Dementia Scale + Profile of Mood States) | Cross-sectional | PH: comorbidity -, depression –  MH: depression - |
| Rueda et al  2011 | Canada, multicenter (unknown) | 361  94% m, 42 ± 9y, 85% Caucasian, 396 ± 263 CD4, 55% undetectable VL (<500 copies) | MOS-HIV  (+ neuropsychological tests) | Cross-sectional | PH, MH: employment +  Employment status had a greater impact on PH than on MH |
| Sarna et al  1999 | USA, multicenter (6) | 33  all females, mean age 34.4 ± 7 y, 14% white, 27% Hispanic, 52% black, 327.9 ± 268.2 CD4 | HOPES | Longitudinal (Baseline, 2 months, 4 months) | HRQoL improved over a 4 month period, married women seemed to be more vulnerable to disruptions in HRQoL over time |
| Schroecksnadel et al  2008 | Austria, monocenter | 152  65.1% m, median 37y | Multidimensional QoL questionnaire for HIV/AIDS  (+ BDI) | Cross-sectional | HRQoL: haemoglobin +, viral load -, depressive symptoms - |
| Sherbourne et al  2000 | USA, multicenter (unknown) | 2864  77% m, mean 39 ± 9 y, 49% white, 33% African American, 15% Hispanic, 49% homosexual, 24% IDU | HCSUS tool (+ Composite International Diagnostic Interview (psychiatric conditions)) | Cross-sectional | PH and MH: probable diagnosis of any mood disorder -,  Heavy drinking was not associated with HRQoL  Probable drug dependence was associated with poorer HRQoL, but most of the effect disappeared after controlling for presence of a probable mood disorder. |
| Soussa et al  1999 | USA, multicenter (3) | 142  80% m, mean 38 ± 8.7y, 56% homosexual, all AIDS or ≤ 200 CD4 | MOS-30  (+ HIV problem checklist (symptoms) + HIV quality audit marker (functional status)) | Cross-sectional | HRQoL: Symptoms -, functional status +, general health perceptions + |
| Szaflarski et al  2006 | USA, multicenter (4) | 449  86% m, 43.3 ± 8.4y 45.2% Caucasian, 50.1% African American, 76.5% on ART | HAT-QoL (+the Duke Religion Index (DUREL), Functional Assessment of Chronic Illness Therapy—Spiritual Well-Being—Expanded (FACIT-Sp-Ex) scale, rief RCOPE (religious coping) + HIV Symptom Index + CES-D) | Cross-sectional | HRQoL (feeling that life has improved): spirituality/religion +, also indirect effects through healthy beliefs and health status/health concerns |
| Tillmann et al  2004 | Germany, monocenter | 250  82.4% m, 40.4 ± 10.6y, 56.4% homosexual, 14.4% IDU, mean VL 34 234 ± 98 438 copies, mean CD4 cell count 328 ± 215 | EuroQol | Cross-sectional | GB virus C coinfected HIV patients had better HRQoL than HIV patients without GB virus C co-infection |
| Trepanier et al  2005 | Canada, monocenter | 155  mean 41.5 ± 8.5, 93% gay/bisexual males, 25% < 200 CD4, 51% undetectable VL (<500), 79% on ART | MOS-HIV (+ Neuropsychological battery + BDI) | Cross-sectional | PH: neuropsychological status +, depression x neuropsychological status interaction, no effect of depression  MH: depression -, no effect of neuropsychological status and the depression x neuropsychological status interaction |
| Uphold et al  2007 | USA, multicenter (3) | 226  all males, 45.7 ± 8.7, 55% white | HCSUS tool (+ Health-Promoting Lifestyle Profile II (HPLP-II) + Psychiatric Epidemiology Research Interview) | Cross-sectional | PH, MH and overall HRQoL: health behaviours +, stress - |
| Worthington  et al  2005 | Canada, monocenter | 308  91% m, mean 41 ± 8 y, 73% homosexual, 9% IDU, CD4 438 ± 286, 82% on ART | MOS-HIV | Cross-sectional | HRQoL: employment +, symptoms - |
| Zinkernagel et al  2001 | Switzerland, multicenter (2) | 318  66% m, median 38y, 403 median CD4 , 2.15 median VL, 78% on ART | MOS-HIV + EuroQol VAS  (+ Hospital Anxiety Depression Scale + State-Trait Anxiety Inventory) | Longitudinal (baseline and every 6 months) | MH: higher education +, being employed +, viral load -, female gender +, HIV duration - |

Multicenter (number) = number of sites, m = males, y = years, VL = viral load, PH = physical health, MH = mental health, + = positive association, - = negative association
