# Supplementary figures and images for: What determines health-related quality of life among people living with HIV: an updated review of the literature
Source: Arch Public Health. 2014 Nov 17;72:40. doi: 10.1186/2049-3258-72-40 (PMC4323115; doi:10.1186/2049-3258-72-40)

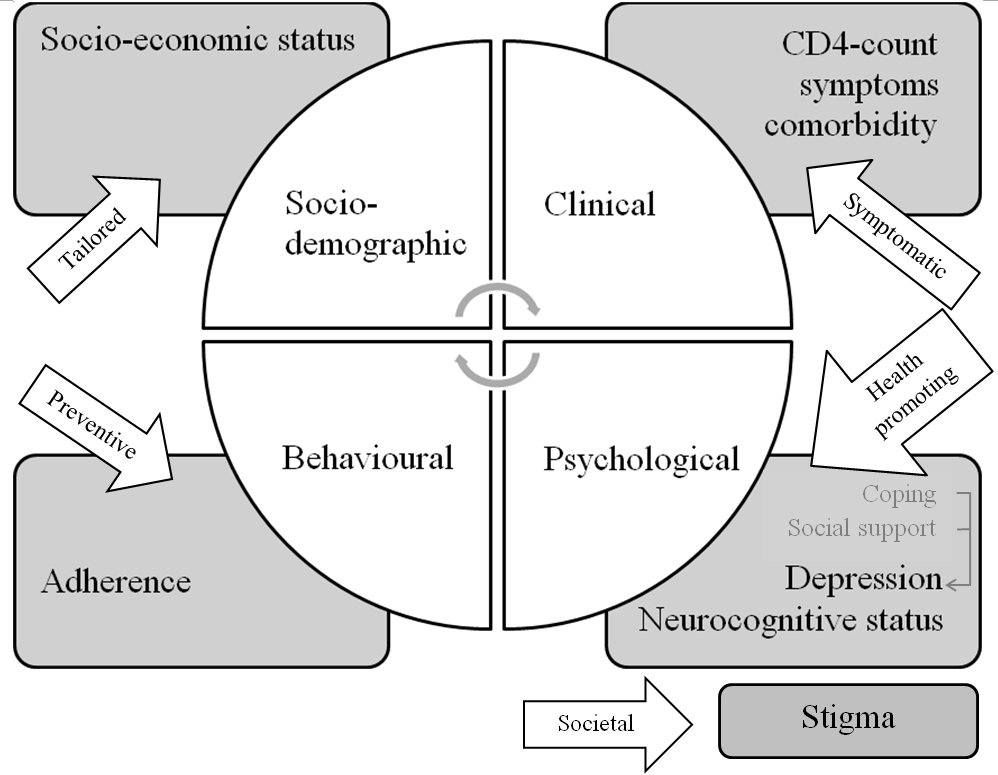

Supplement: Supplementary file 2 — Authors’ original file for figure 1 [file 13690_2014_5055_MOESM2_ESM.bmp]
